# Thoracic Aortic Aneurysm Presenting as a Subacute Cough

**DOI:** 10.24908/pocus.v8i1.15894

**Published:** 2023-04-26

**Authors:** Eduardo Diaz, Hanan Atia, Brian Kohen, Seth Lotterman

**Affiliations:** 1 Emergency Medicine Residency, Memorial Healthcare System Pembroke Pines, FL USA; 2 Associate Director of Emergency Medicine & Core Clinical Faculty, Memorial Healthcare System Pembroke Pines, FL USA; 3 Director of Emergency Ultrasound & Core Clinical Faculty, Memorial Healthcare System Pembroke Pines, FL USA; 4 Department of Emergency Medicine, Hartford Hospital Hartford, CT USA

**Keywords:** aortic aneurysm, Suprasternal aortic view

## Abstract

The suprasternal aortic notch cardiac point of care ultrasound (POCUS) window is a useful view for evaluating thoracic aortic pathologies. However, it is not routinely included in the standard cardiac POCUS exam despite its ability to capture emergent pathologies such as aortic dissection and thoracic aortic aneurysm (TAA). Ruptured aortic aneurysms can present with sudden, severe chest or back pain, as well as hemodynamic instability, resulting in a high mortality. We present an atypical case of a patient with hemoptysis who was found to have a contained aortic rupture. In this case, POCUS, specifically the suprasternal aortic notch view, was used to expedite definitive care.

## Case

A 33-year-old female with a past medical history of obesity and untreated hypertension presented to the emergency department (ED) with hemoptysis that began one day prior to presentation. The patient reported one week of a dry cough that preceded the hemoptysis. Symptoms associated with hemoptysis included shortness of breath, rhinorrhea, and a generalized headache. She denied chest pain, back pain, pleurisy, numbness, weakness or paresthesias. 

On presentation, the patient’s vital signs were BP 168/126 mmHg, HR 123 bpm, RR 26 breaths/minute, temperature 37.3 °C, and SpO2 97% on room air. On physical examination, she appeared comfortable and in no acute distress. Her lungs were clear to auscultation bilaterally, and there were no murmurs, rubs or gallops.

**Figure 1:**
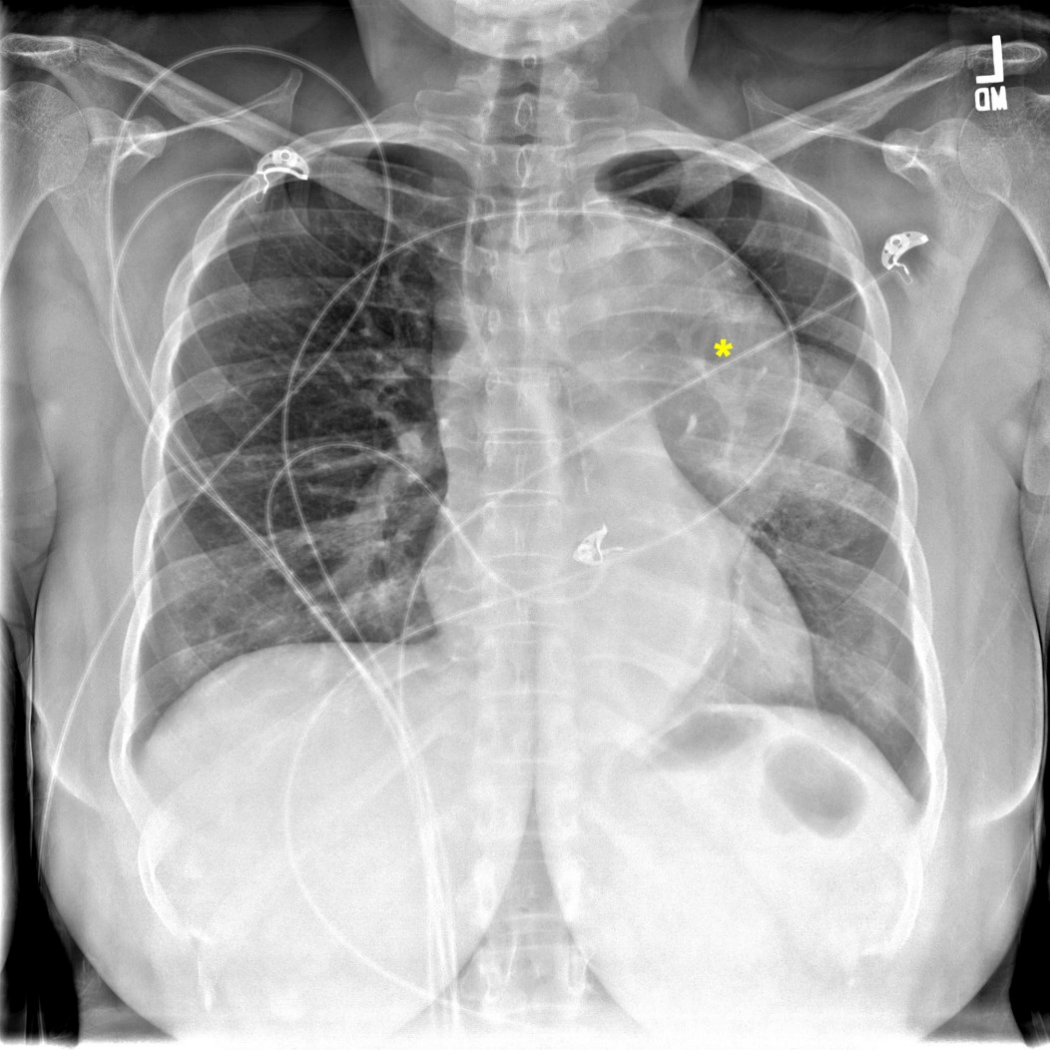
Anteroposterior view chest X-ray showing a mediastinal lesion (*).

**Figure 2:**
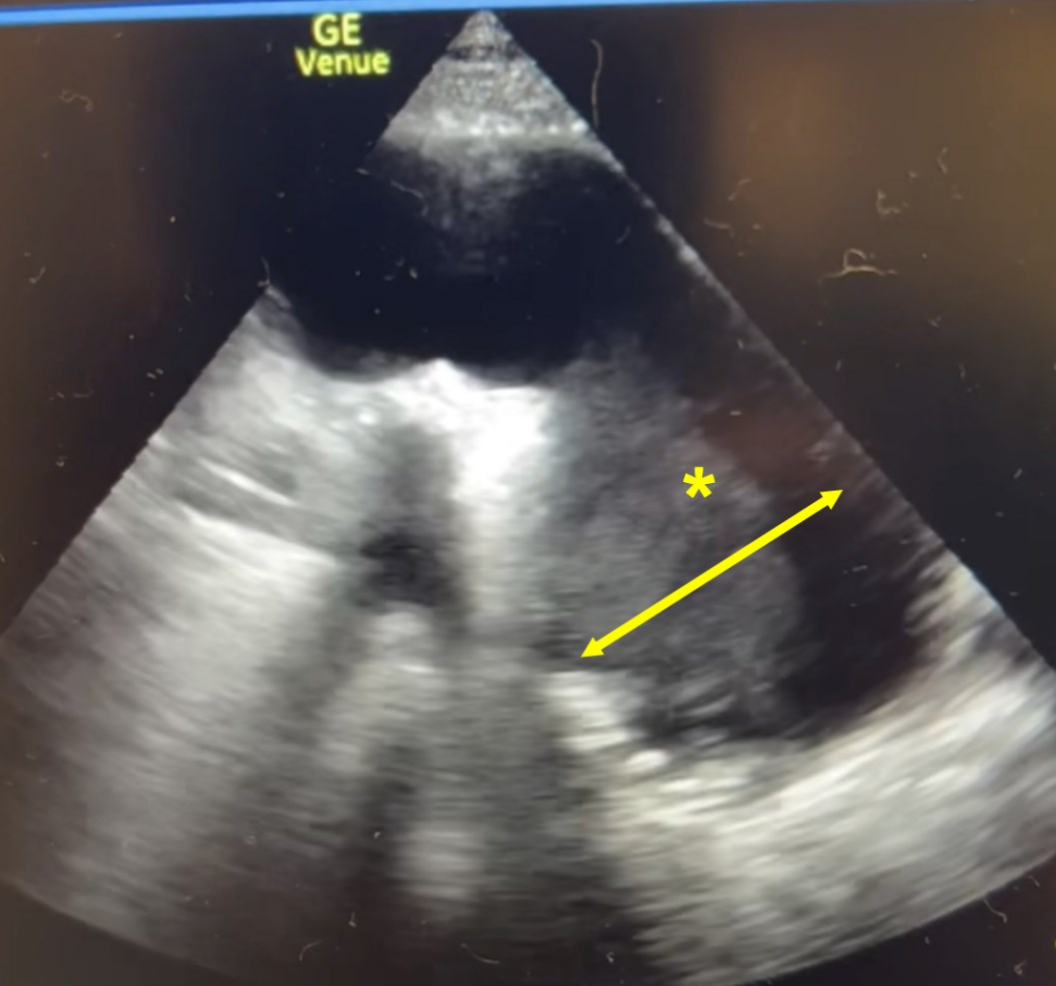
Suprasternal aortic notch POCUS showing a hyperechoic lesion (*) in a dilated descending aorta.

**Figure 3:**
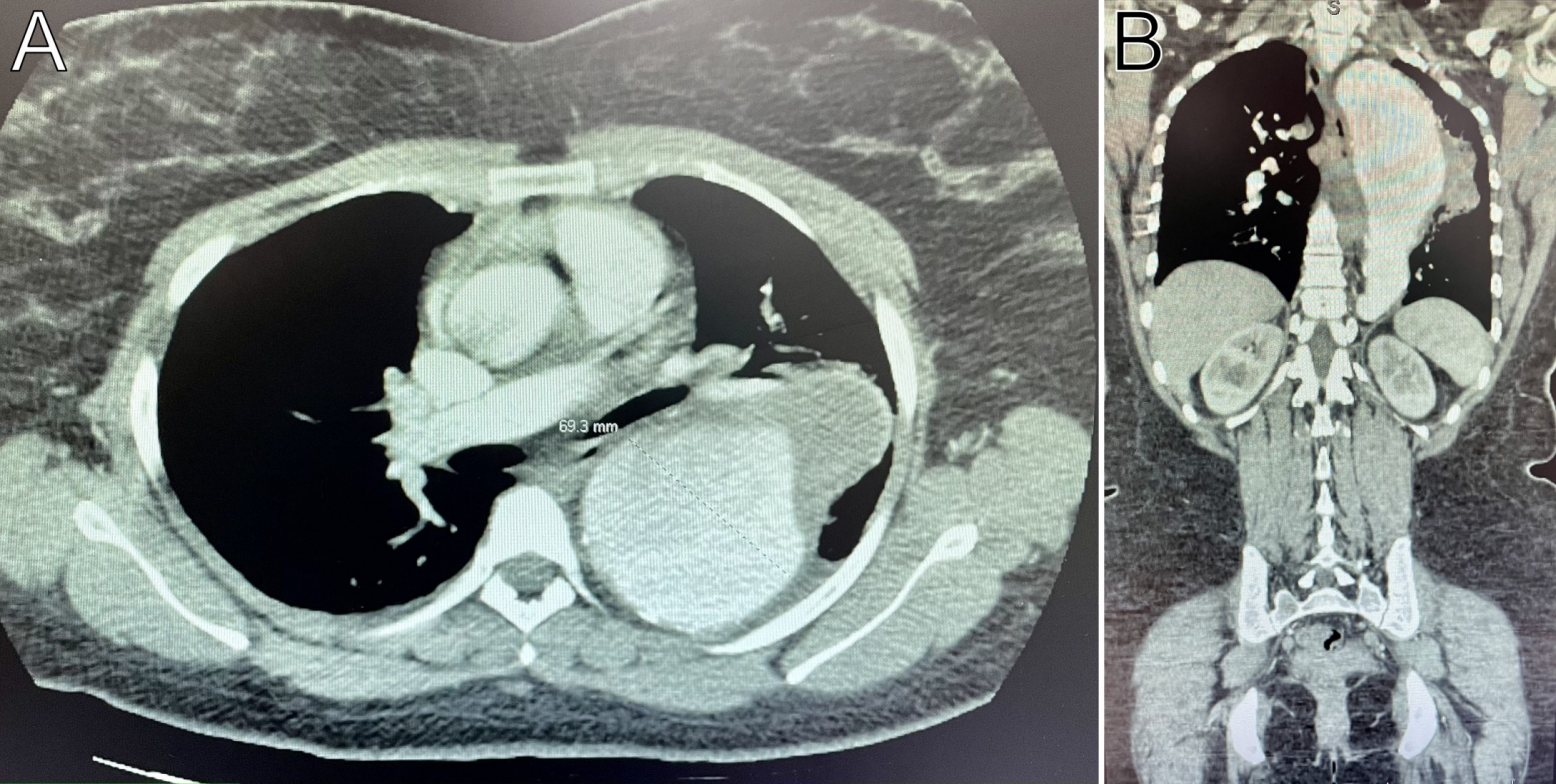
Computed tomography angiogram of the aorta showing a descending thoracic aortic aneurysm and contained aortic rupture. A) the aneurysm in the axial plane, B) the aneurysm in the coronal plane.

A chest X-ray was ordered and interpreted by radiology to be concerning for a left-sided mediastinal mass in the area of the thoracic aorta (Figure 1). To further evaluate the proximal aorta given the high clinical suspicion for pathology, a suprasternal aortic notch point of care ultrasound (POCUS) was performed demonstrating a dilated descending thoracic aorta with a contained mobile hyperechoic collection (Figure 2). Given the POCUS findings, the patient was emergently sent for a computed tomography angiogram of the aorta which revealed a 7 cm descending thoracic aortic aneurysm (TAA) with irregularity of the anterior lateral wall suspicious for a contained rupture (Figure 3). The patient was subsequently admitted to the cardiovascular intensive care unit and underwent thoracic endovascular aortic repair.

## Discussion

The American Institute of Ultrasound Medicine recommends five standard views for the cardiac POCUS exam, including parasternal long and short axes, apical four-chamber, subxiphoid cardiac, and inferior vena cava 1. In the parasternal long axis and the apical four-chamber, only a single cross-sectional image of the descending aorta is visualized, limiting its diagnostic capability for aortic pathology. In the suprasternal aortic arch view, the aortic arch and its branches can be visualized in a long axis, allowing more thorough assessment of the aorta. The suprasternal aortic notch view is often omitted, likely because of its difficulty to acquire due to probe size, patient habitus, and mobility. Additionally, this view is mainly used to identify TAA and aortic dissection, life-threatening but rare conditions 2. Other non-emergent applications of this view include evaluation for aortic arch plaques and coarctation of the aorta. A 2009 study in the European Journal of Echocardiography found that out of 2,000 adult patients sent for transthoracic echocardiography, 2% had abnormal aortic arch pathology that led to therapeutic intervention 3. As demonstrated in this case, the suprasternal aortic notch view provided us with information that allowed us to expedite proper intervention for the patient. 

**Figure 4:**
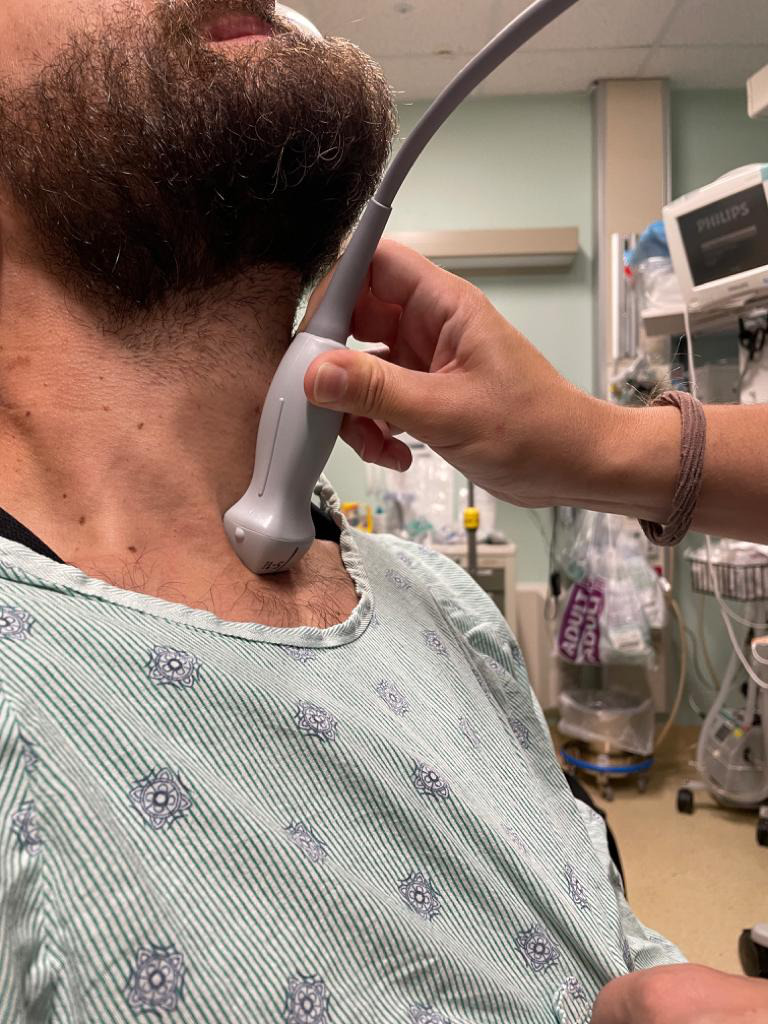
Positioning for the suprasternal aortic notch POCUS window with the patient’s neck extended and the probe marker pointing towards the patient’s right shoulder.

**Figure 5:**
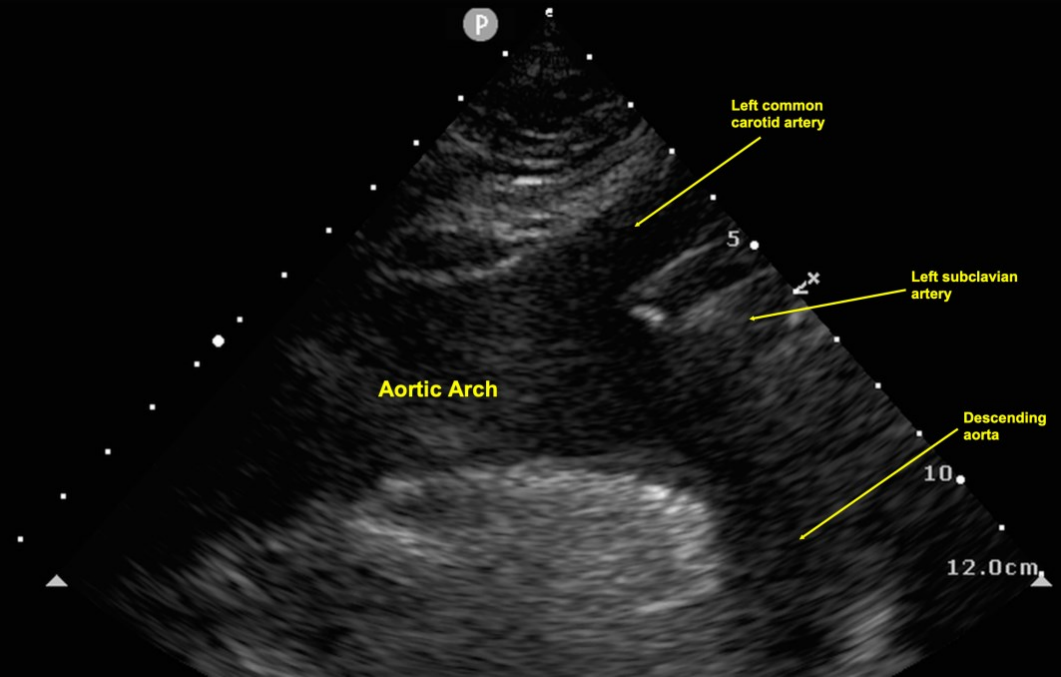
Normal aortic arch anatomy as seen on a suprasternal aortic notch POCUS. Note, this is the emergency medicine convention for probe and screen marker, not the echocardiography convention.

To obtain the suprasternal aortic notch view, position the patient supine with full neck extension. A pillow can be placed in between the shoulders to further exaggerate neck extension. A generous amount of ultrasound gel should be used to avoid applying excess pressure on the patient’s neck. In some patients with limited neck extension, turning the head to the side is helpful. Once optimal positioning is obtained, using the phased array probe, point the probe marker towards the patient’s right shoulder (in emergency medicine cardiac POCUS convention; Figure 4). In this view, the aortic arch with its three branches can be visualized. From proximal to distal, these include the brachiocephalic, left common carotid, and subclavian arteries (Figure 5). 

## Conclusion

TAA, though uncommon, is a life-threatening condition given the increased risk of rupture 4, 5. We presented an unusual case of a 33-year-old female who was evaluated for new onset hemoptysis in the context of a subacute cough. The suprasternal aortic notch POCUS window was an integral aspect of the patient’s evaluation, allowing us to expedite advanced imaging, surgical consultation, and definitive treatment. ED providers should consider incorporating the suprasternal aortic notch view if there is a high clinical suspicion for thoracic aortic pathology. 

## Ethics Approval

According to Memorial Healthcare System’s policies, publication of a single case report or cases series involving data from three or fewer patients, and that does not involve an investigation of an FDA-regulated product is not considered research but an educational activity and does not require IRB review. Such presentations should reflect the personal experience of a presenter and not the result of formal clinical research.

## Conflicts of Interest

None.
